# Central Precocious Puberty as a Complication of Therapy with Adrenocorticotropin (ACTH) and an Aromatase Inhibitor for Refractory Nephrotic Syndrome

**DOI:** 10.1155/2019/1624274

**Published:** 2019-04-04

**Authors:** Sungeeta Agrawal, Robert Gensure, Lawrence Milner, Julie Nicoletta, Abdollah Sadeghi-Nejad

**Affiliations:** ^1^Division of Pediatric Endocrinology, Floating Hospital for Children, Tufts Medical Center, Tufts University School of Medicine, Boston, MA, USA; ^2^Division of Pediatric Nephrology, Floating Hospital for Children, Tufts Medical Center, Tufts University School of Medicine, Boston, MA, USA

## Abstract

Glucocorticoids are typically prescribed for the treatment of idiopathic nephrotic syndrome of childhood. In selected patients with refractory focal segmental glomuerulosclerosis (FSGS), adrenocorticotropin (ACTH) can be used to induce remission and decrease the progression of the disease. We report a 6 8/12-year-old girl with recurrent proteinuria, resistant to standard immunotherapy. She underwent related renal transplant but again developed proteinuria and was started on ACTH. She subsequently developed peripheral precocious puberty (PPP), presumably from peripheral aromatization of adrenal androgens. She was started on an aromatase inhibitor, and her ACTH dose was slowly decreased. She then developed central precocious puberty (CPP). We hypothesize that treatment of her peripheral precocious puberty with an aromatase inhibitor may have triggered central precocious puberty.

## 1. Introduction

Development of secondary sexual characteristics is considered early if it occurs before age 8 years in girls and before 9 in boys [1, 2]. The disorder is broadly divided into two categories. Patients with central precocious puberty (CPP) have early maturation of the hypothalamic-pituitary-gonadal axis resulting in increased gonadal hormones and secondary sex development. In contrast, those with peripheral precocious puberty (PPP) have early secondary sex development and high concentrations of sex hormones, but the hypothalamic-pituitary-gonadal axis is suppressed. We report a young girl who developed PPP during treatment with an adrenocorticotropin (ACTH) analog (Acthar gel) for refractory nephrotic syndrome, presumed to be caused by focal glomerular sclerosis (FSGS). Subsequently, after a decrease in ACTH gel and treatment with an aromatase analog, she developed CPP.

## 2. Case Report

This 6 8/12-year-old Hispanic girl developed nephrotic syndrome at age 2 7/12 years, which was found to be steroid resistant. Renal biopsy showed minimal changes with focal IgM staining. Proteinuria continued despite treatment with steroids and immunosuppressive drugs, and repeat renal biopsy showed significant focal glomerular sclerosis (FSGS). She continued to have marked proteinuria and edema. She was also growing at <3 percentile for height. With the prospect of a need for transplantation, treatment with growth hormone was discussed with the family but they declined. Renal function deteriorated rapidly, and she was started on continuous cycled peritoneal dialysis for one year. She had a kidney transplant from her mother at age 3 9/12 years.

She developed proteinuria immediately after transplant (day zero), which continued despite aggressive medical therapy with immune-suppressants, plasmapheresis, and rituximab. Six months later (age 4 3/12 years), she was started on an ACTH analog (Acthar gel 40 units IM three times a week). There was gradual resolution of her proteinuria and edema over the next few months. She remained in remission on ACTH. However, she developed obesity and a generalized increase in body hair. She continued to have poor linear growth, which was attributed to familial factors, her renal disease, and ACTH therapy.

Almost three years after transplant, at age 6 8/12 years, she was seen by endocrinologists for concerns of poor growth and possible precocious puberty. On exam, she was Cushingoid. She was 109 cm tall (2.2% tile), weighed 22.9 Kg (61% tile), and body mass index was 19.3 (95% tile). She had generalized increase in body hair and a small amount of axillary hair. She was Tanner 3 for breast development and Tanner 4 for pubic hair. The clitoris was normal, and the vaginal mucosa appeared to be prepubertal. Laboratory studies confirmed high concentrations of cortisol and adrenal androgens (Table 1, Month 0). Gonadotropins were at prepubertal levels, and estradiol was elevated. Bone age was only mildly advanced at 7 10/12 at chronologic age of 6 8/12 years. Likewise, pelvic ultrasound showed prepubertal uterus with imperceptible endometrial stripe (<1 mm), right ovarian volume 0.8 cc, and left ovarian volume 1.3 cc. Additional biochemical studies are also shown in Table 1.

Our impression was that ACTH treatment had resulted in hypersecretion of adrenal androgens and that breast development was the result of aromatization of androgens. There were concerns that a rapid reduction in ACTH dose might result in recurrence of proteinuria. Accordingly, her ACTH dose was slowly decreased. Two months after initial presentation, due to continued breast development, she was started on an aromatase inhibitor (anastrozole 0.5 mg daily) to block the conversion of androgens to estrogen (Table 1, Month 2).

While on the aromatase inhibitor, her breast tissue regressed, and she was Tanner 2 on repeat exam 1 month after starting the medication. Over the next month, however, breast development resumed while still on treatment with aromatase inhibitors, and laboratory studies showed increasing concentrations of gonadotropins and estradiol (Table 1, Month 4). A repeat pelvic ultrasound now showed maturing ovaries and uterus, all consistent with development of central precocious puberty. She was then started on an analog of luteinizing hormone-releasing hormone (LHRH; depo-lupron 30 mg IM once every 3 months) to suppress gonadotropin production. After 4 months of LHRH therapy, she has had suppression of gonadotropins (Table 1, Month 8) and resolution of breast development. Over one year after starting LHRH therapy, her puberty is still suppressed, both clinically and biochemically (Table 1, Month 20). Her interval linear growth has been appropriate, and her nephrotic syndrome is still in remission.

## 3. Discussion

Glucocorticoids remain the mainstay of treatment of nephrotic syndrome, with newer immunotherapeutic drugs used in addition. ACTHar gel, prepared from porcine pituitary glands, contains ACTH and other POMC peptides [3]. Its use is now reserved for treatment of steroid-resistant nephrotic syndrome. The mechanism by which ACTH improves proteinuria is not clear though studies have found melanocortin receptors (receptors of ACTH) in the kidney [3, 4]. One study found that, in patients with FSGS, treatment using ACTHar gel alone led to complete remission in 7.7% of patients and partial remission in 62% of patients [5]. Our patient's proteinuria resolved after starting ACTH therapy even though she was resistant to combined immunosuppressive therapy and plasmapheresis.

The common side effects of ACTH treatment are similar to those seen with glucocorticoids, such as weight gain, Cushingoid facies, fluid retention, hyperglycemia, hypertension, hypokalemia, and increased irritability [3, 5, 6]. Chronically elevated ACTH levels can also lead to excess adrenal androgens, which can in turn lead to increased estrogen [7] and gonadotropin suppression [8]. On the other hand, studies in adult women show ACTH administration can stimulate LH and FSH release, possibly through cortisol [9]. Side effects of ACTH used to treat nephrotic syndrome are not well documented in children due to infrequent use; however, ACTH is also used more commonly to treat infantile spasms and intractable epilepsy in children. In this group, hypertension was found to be the most common adverse effect in one study, followed by weight gain [10].

Peripheral precocious puberty is seen after exposure to exogenous gonadal steroids or gonadotropin-independent secretion of androgens or estrogens. In the former, treatment is the removal of the exogenous hormone. In the latter group, treatment aims at medical or surgical stoppage of hormone production. In this patient, we believe PPP was the result of increased adrenal androgens (caused by ACTH) and their conversion to estrogen.

Whether or not PPP can induce CPP is controversial. CPP has been reported in children after excision of adrenal adenoma and Leydig cell tumor of the testicle [11, 12]. CPP has also been seen after treatment of congenital adrenal hyperplasia, far more than in the general population [13–15]. The exact mechanism by which CPP could develop after treatment of PPP is not known. It is postulated that elevated levels of testosterone lead to early maturation of the gonadotropin-releasing hormone (GnRH) pulse generator [11, 14]. Subsequent reduction of androgen/estrogen levels results in CPP. Consistent with this hypothesis, an increased incidence of CPP was observed in a series of patients treated for familial male-limited testotoxicosis with sprinolactone and an aromatase inhibitor [16].

It has also been suggested that increased weight and advanced bone age may result in early maturation of the LHRH pulse generator [11, 15]. Our patient was obese and had high concentrations of adrenal androgens (though her bone age was only one year advanced). Lowering her ACTH dose resulted in decreased production of androgens. In addition, the aromatase inhibitor decreased their conversion to estrogens. The decreased levels of androgen and estrogen can result in disinhibition of the hypothalamic-pituitary-ovarian axis, which had previously been activated by the weight gain.

This patient developed exogenous Cushing syndrome and PPP after treatment with ACTH for refractory nephrotic syndrome. To our knowledge, development of CPP after ACTH therapy has not previously been reported. Furthermore, findings in our patient support the idea that treatment of PPP with anastrazole can unmask CPP. We conclude that prepubertal children treated with ACTH should be monitored for development of PPP and CPP.

## Figures and Tables

**Table 1:** Laboratory test results and medication dose at different time intervals.

	Reference range	Month 0^a^	Month 1^b^	Month 2^c^	Month 4^d^	Month 8^e^	Month 20^f^
Treatment	n/a	—	—	Anastrozole	Anastrozole, lupron	Anastrozole, lupron	Anastrozole, lupron
LH (mIU/ml)	<0.2	0.12	1.4	4.7	1.7	0.5	0.3
FSH (mIU/ml)	1–4.2	1.7	2.6	7.5	5.5	0.6	0.8
Estradiol (pg/ml)	5–20	12.6	<10	42.9	67.3	<10	<10
Testosterone (ng/dl)	<2.5–10	—	36	24	35	25	<13
Cortisol AM (mcg/dl)	3–21	69.1	6.1^*∗*^	4.2	2.6	5.6	1.2
17-OHP (ng/dl)	<91	71	—	32	24	9	<0.8
DHEA (ng/dl)	19–592	1096	—	—	206	177	167
DHEAS (mcg/dl)	<92	396	—	268	194	142	150
Androstenedione (ng/dl)	6–115	—	—	188	229	143	18

^*∗*^We attribute the apparent rapid fall of cortisol due to timing of sample in relation to ACTH administration. ^a^ACTH 40 units three times/week. ^b^ACTH 40 units twice/week and 32 units once/week. ^c^ACTH 40 units once/week and 32 units twice/week, started anastrozole 0.5 mg/day. ^d^ACTH 32 units three times/week, anastrozole 0.5 mg/day, started LHRH 30 mg once/3 months. ^e^ACTH 24 units twice/week, anastrozole 0.5 mg/day, LHRH 30 mg IM once/3 months. ^f^ACTH 8 units once/week, prednisolone 3 mg six times/week, anastrozole 0.5 mg/day, LHRH 30 mg IM once/3 months.

## Abbreviations

FSGS: Focal segmental glomuerulosclerosis
ACTH: Adrenocorticotropin
PPP: Peripheral precocious puberty
CPP: Central precocious puberty
LHRH: Luteinizing hormone-releasing hormone.

## References

[1] Marshall W. A., Tanner J. M. (1969). Variations in pattern of pubertal changes in girls. *Archives of Disease in Childhood*.

[2] Marshall W. A., Tanner J. M. (1970). Variations in the pattern of pubertal changes in boys. *Archives of Disease in Childhood*.

[3] Gong R. (2011). The renaissance of corticotropin therapy in proteinuric nephropathies. *Nature Reviews Nephrology*.

[4] Cone R. D. (2006). Studies on the physiological functions of the melanocortin system. *Endocrine Reviews*.

[5] Tumlin J., Galphin C., Santos R., Rovin B. (2017). Safety and efficacy of combination ACTHar gel and tacrolimus in treatment-resistant focal segmental glomerulosclerosis and membranous glomerulopathy. *Kidney International Reports*.

[6] Berg A.-L., Nilsson-Ehle P. (1996). ACTH lowers serum lipids in steroid-treated hyperlipemic patients with kidney disease. *Kidney International*.

[7] Higashi S., Aizawa Y. (1980). Effect of ACTH on adrenal estrogens. *The Japanese Journal of Pharmacology*.

[8] Bertagna X. (2017). Effects of chronic ACTH excess on human adrenal cortex. *Front Endocrinol (Lausanne).*.

[9] Aleknaviciute J., Tulen J. H. M., Timmermans M. (2016). Adrenocorticotropic hormone elicits gonadotropin secretion in premenopausal women. *Human Reproduction*.

[10] Angappan D., Sahu J. K., Malhi P., Singhi P. (2018). Safety, tolerability, and effectiveness of oral zonisamide therapy in comparison with intramuscular adrenocorticotropic hormone therapy in infants with West syndrome. *European Journal of Paediatric Neurology*.

[11] Ghazi A. A., Rahimi F., Ahadi M. M., Sadeghi-Nejad A. (2001). Development of true precocious puberty following treatment of a leydig cell tumor of the testis. *Journal of Pediatric Endocrinology and Metabolism*.

[12] Ersoy B., Kizilay D., Cayirli H., Temiz P., Gunsar C. (2017). Central precocious puberty secondary to adrenocortical adenoma in a female child: case report and review of the literature. *Journal of Pediatric and Adolescent Gynecology*.

[13] Witchel S. F. (2017). Congenital adrenal hyperplasia. *Journal of Pediatric and Adolescent Gynecology*.

[14] Pescovitz O. H., Comite F., Cassorla F. (1984). True precocious puberty complicating congenital adrenal hyperplasia: treatment with a luteinizing hormone-releasing hormone analog. *Journal of Clinical Endocrinology & Metabolism*.

[15] Dacou-Voutetakis C., Karidis N. (1993). Congenital adrenal hyperplasia complicated by central precocious puberty: treatment with LHRH-agonist analogue. *Annals of the New York Academy of Sciences*.

[16] Leschek E. W., Jones J., Barnes K. M., Hill S. C., Cutler G. B. (1999). Six-year results of spironolactone and testolactone treatment of familial male-limited precocious puberty with addition of deslorelin after central puberty onset. *Journal of Clinical Endocrinology & Metabolism*.

